# Market conditions of international VET providers: a comparative analysis of Australia, UK, USA, and Germany

**DOI:** 10.1186/s40461-021-00128-w

**Published:** 2021-12-18

**Authors:** Susanne Peters

**Affiliations:** Institute Technology and Education (ITB), University of Bremen, Am Fallturm 1, 28359 Bremen, Germany

## Abstract

VET (vocational education and training) is a highly complex, multidimensional worldwide phenomenon with diverse structures. Additionally, very different actors define the functions of a national (or even a regional) VET system. The paper contributes to a better understanding of the policy frameworks and current states of such systems. Therefore, we focus on selected VET systems in order to understand their specifics and thus, their market conditions. A qualitative approach is used to answer the research question regarding which conditions create or support market-based opportunities for the provision of commercial vocational training services. We find that the liberalism and deregulation of the VET sectors, as well as the marketisation of VET practices, lead to incentives to internationalise VET offers. Thinking in terms of skills, the kind of education system does not play a role. This is the case in liberal market-driven VET approaches (here, the UK, the USA and Australia) and is mirrored in the micro-analysis categories of *curricula*, *learning location*, *content*, and *learning process*.

## Introduction

In Germany, vocational education is popular and enjoys a good reputation. German vocational training, especially dual vocational training, is considered to be an “export hit” from a German perspective and a guarantee of high quality and reliability. The reasons for this are its effective influence on economic and social development, not least on youth unemployment (Bohlinger and Wolf 2016). The dual approach has also been attracting a great deal of attention from international and supranational organisations for at least a decade (ILO 2021; OECD 2011). Furthermore, a demand from abroad for vocational training “Made in Germany” can be observed (Hilbig and Nirenberg 2019; iMove 2017; Kühn 2020).

The possibilities for Vocational Education and Training (VET; here also used interchangeably with TVET, Technical Vocational Education and Training) providers when approaching a new market are manifold. A common feature here is the development of a business model that is oriented to the provider’s own resources on the one hand and to the requirements of the market on the other hand (for example, see Bullinger and Scheer 2003; Leimeister 2020; Osterwalder and Pigneur 2013). Still, there are only a few German education providers that offer VET on international markets – although it stands to reason, that Germany is at the forefront of knowledge and competences in terms of VET. One successful producer is Festo Didactics,^1^ one of the world’s leading service providers in the field of technical education. Festo sells technical infrastructure for VET, and therefore, the business model serves a very specific segment and not the broad spectrum of vocational education. Another example is Udacity,^2^ but here, vocational education is rather a by-product, as Udacity offers no original VET but online programmes instead.

We wonder why the profit potential of German providers – with their expertise – is not being exploited and wish to investigate German TVET providers and their strategies by comparing them to Anglo-Saxon international providers and their equivalent strategies. To answer this question, the term of interest is the *market condition* of VET providers. But first, a clarification of the term *VET providers* is helpful: In this research, VET providers are private, profit-oriented suppliers on the training market. The products include initial, continuing or sector-based vocational training offers.

In this study, we compare different education and training systems in selected countries (Australia, USA, UK and Germany) to get starting points that explain how private education providers position themselves in the markets. Australia, the United Kingdom and the United States are the countries of interest to be compared to Germany. We chose these three countries due to the following criteria: Firstly, all of them have a high activity level in the technical and vocational education and training market (BMBF 2000). Secondly, we would like to consider different continents and economic regions, so here we have included North America, Europe, and Australia through the sample of countries. Also, and thirdly, TVET certificates should be recognised as important—this is the case in all the chosen countries. Furthermore, findings of a survey by iMOVE (2017) point out that international TVET providers consider Australia, the UK and the USA to be the most important and most successful markets. Other researchers consider these three countries to be global pioneers in terms of economic sales of vocational training (Fraunhofer MOEZ 2012).

The provision of private training offers is, in traditional VET systems like Germany, a complementary option to state training offers. This article seeks not to regard developments at the systemic, state level of vocational education. Still, we argue that VET systems and their specifics create market conditions. Our assumption is that different frameworks support the successful supply of VET services. For example, there are huge divisions between public and private education funding (Busemeyer and Iversen 2014). In countries with a high state provision of education, there is no need for a strong private education system – as long as the quality is accepted by the population. So, the share of public education programmes and private investment in education could be an indicator for existing markets.

Several researchers show first insights to the market conditions of VET providers. Pilz and Wiemann (2020) show different internationalisation strategies of VET providers according to their organisational orientation and their objectives related to the target market. The study describes the strategies based on the closeness or distance between the dual model and the target market (ibid.); the transfer processes are not within the scope of the research. Korbel and Misko (2016) analyse the Australian VET provider market structure: Policy changes over the last two decades have led to incremental but increasing competition. Indicators, like provider registration patterns and comparisons to higher education, point to the conclusion that there might be an imbalance in Australia regarding the number of providers and given the distribution and demographics of the labour force. Toner (2018) describes Anglo-Saxon training markets developments in the last 30 years, mainly led by the principle of competition between public and private providers. According to the author, VET providers are defining their competitive advantage by tailoring their offerings to specific customer segments (ibid.). Except for prices and convenience (e.g. course durations) these adjustments are not further specified.

Regarding market conditions of VET providers, an issue related to the business model developments of these providers should be highlighted. Business models in the educational sector are a new research field and are becoming more important because of an increase in online learning platforms, learning apps and management learning systems (Hilbig and Nirenberg 2019; Kusainov et al. 2021). These methods of learning are changing the ways that training providers create, deliver and capture value in different markets. The relationship between a provider and its customer is based on the so-called double customer logic when regarding private vocational training providers: Learners are direct customers and are actively involved in receiving educational services, but the client who books and pays for the training is mostly someone else, e.g. a company, administrative institution or government. For that reason, in many cases, vocational training providers are dealing with two different customers, both of which have to be satisfied (Hilbig and Nirenberg 2019).

These first insights into the market conditions of VET providers are helpful, yet they do not reveal to what degree vocational training providers can innovate, adapt or modify their business models to seize the foreign business opportunities in order to gain competitive advantage (Hilbig and Nirenberg 2019). So, what are the necessary factors and conditions for developing competitiveness?

**Fig. 1:**
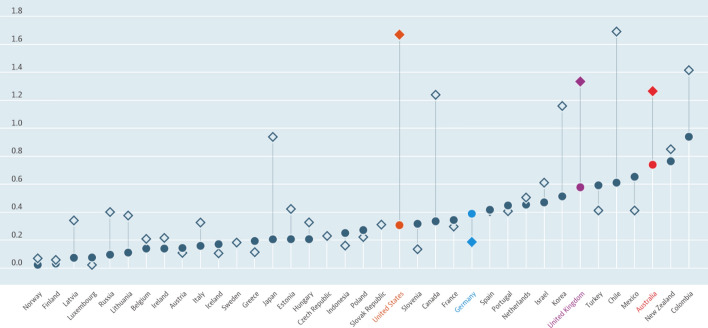
Private spending on education. ● Primary to post-secondary non-tertiary/♦ Tertiary, % of GDP, 2016 or latest available. https://data.oecd.org/eduresource/private-spending-on-education.htm

We argue for an interrelation between the customers’ willingness to pay for education and the structure of the public education opportunities. Figure 1 shows the level of private investment in education and training, divided into tertiary education and primary to secondary education, as percentages of GDP. We can see huge differences between Germany and the sample countries regarding their expenditure on education and educational services, especially in the tertiary education sector. Moreover, more than 60% of funding on tertiary institutions is privately sourced in Australia, the United Kingdom and the United States, for example. These countries also tend to charge students higher fees (OECD 2019). A large supply of commercial offers in this field could imply a higher willingness of potential customers from private households to pay for educational services in the Anglo-Saxon countries. In Germany, the situation is different. Here, customers are oriented towards public education, and therefore, they are not ready to use and pay for private education institutions and training offers. However, despite this apparent structural cause, which will be discussed later, there is a market for commercial educational services, as the existence of providers shows.

Our research questions, coming from our assumption that the conditions create or support market-based opportunities for the provision of commercial vocational training services, are as follows: Which conditions and characteristics can be determined regarding the markets of VET-related services, with a special regard to Anglo-Saxon countries in comparison to Germany? Which success factors can be derived for VET providers in international markets?

A qualitative approach is useful here as there seem to be structural causes, but only little research exists in this field (see Hilbig 2019). So, in order to conduct a market analysis focusing on the current situation of TVET providers and their practices, we collected empirical data from selected companies. In the method section below, we describe the case selection and research process.

## Theoretical framework and methods

We used Kuckartz’ (2019) general workflow of a qualitative content analysis, or more precisely, the thematic analysis according to Kuckartz (2010). In this study, we thematically analyzed the material deductively in a first step. In the second phase, we reworked it inductively (Peters 2019).

Intensive desktop research and the evaluation of literature leads us to the countries of interest: Australia, the United Kingdom and the United States (see “Introduction”).

We focused on VET providers with regard to the research questions. Rough criteria for the selection of the first interview partner was, that the provider offers educational products in international markets and includes TVET services within these. The other interview partners were chosen based on the following criteria (most different research design, Georg 2005):

1. *Different head office locations.* Companies from different countries and continents including Germany, the US and the UK, ensured that different cultural and social perspectives were included in the sample.^3^
2. *Different company sizes.* Ranging from micro-entrepreneurs and start-ups to SMEs and global market leaders, we interviewed all sizes of companies.
3. *Different educational products.* University courses, train the trainer courses, publishing providers, digitised education and training, VET, initial education and further education belong to the spectrum of services which are offered by our interview partners.
4. *Different positions in the company.* Sales managers, sales directors, managing directors, and company founders were all interviewed.

Finally, to include a contrasting view, we interviewed one German VET provider who does not offer products internationally (yet).

A qualitative approach was applied by contacting a first expert to conduct expert interviews (Bogner et al. 2009). With the aim of data saturation, data were then collected repeatedly. In the respective interviews, new experts were suggested or introduced. Therefore, the experts contacted were defined by the other experts in the field themselves, until theoretical saturation was reached. Consequently, nine providers were willing to share their knowledge with us. Before the interviews, we elaborated on the basics of the VET systems and the export potentials through an extensive literature research. Then, from May 2020 to September 2020, we conducted telephone and Zoom interviews with the following enterprises (Table 1):

**Table 1:** Enterprises and characteristics. Own compilation

Company	Country	Size of company^a^
CityandGuilds	U.K	Large
Pearson	U.S.A	Large
SMG Educational Solutions	Germany	Small
IntAmt	Germany	Small
brainshuttle GmbH	Germany	Micro
SAP training	U.S.A	Large
Klett	Germany	Large
BTEC	U.K	Large
Alfaview	Germany	Medium

In the following, the interview partners quoted anonymously

^a^According to the OECD definition, micro-enterprises are defined as firms with 1–9 persons employed; small enterprises: 10–49; medium enterprises: 50–249; and large enterprises: 250 and more (OECD 2015).

We recorded, transcribed and analysed all the interviews and then applied a qualitative content analysis approach (Kuckartz 2019).

The following discrepancy made us start the analysis at the micro level in the first instance: There are not only respective national definitions of VET, but also numerous subsystems. In addition, components of initial vocational training are often shifted to the area of continuing education: “Given the number of VET programmes which do not even have ‘vocational’ in their title (as with much of further education in England) it often happens in such narratives that the term ‘vocational’ is not used at all “ (CEDEFOP 2017, p. 8). Pilz’s (2016) typology in comparative vocational education provides a theoretical basis in comparative VET research for the analysis of micro-level products. Vocational pedagogical and teaching–learning theoretical analyses were thus incorporated into the research approach in the first step. Therefore, for the deductive analysis, Pilz’ (2016) typology was used and, more precisely, the categories of *curricula*, *learning location*, *content* (e.g. the preparation of teaching content; complex situation learning), and *learning process* (e.g. media and self-directed learning; interaction between teachers and learners). To put it differently, Pilz’ typology was the basis for the analysis of very differentiated and customised VET products at the micro level. It can be seen as the lowest common denominator in VET, which is a highly complex, multidimensional worldwide phenomenon with diverse structures (Pilz and Li 2020).

The aforementioned categories were used in a first round of coding of all the transcribed interviews. Afterwards, we coded all the material again inductively, led by the research questions.

## Diverse structures of VET markets in Germany, Australia, the USA and the UK

Based on theoretical considerations, we argue that the market conditions are different in the sample countries compared to Germany. This includes the conditions in the public vocational education system as well as export potentials. We present the findings of the analysis below.

### Australia

Since the 1990s, the Australian vocational education and training system has been in a state of upheaval, resulting in a system that has become more geared towards the needs of the economy. Consequently, the relevance of VET learning times has been reduced, and the National Strategy Paper on Vocational Education and Training 2004–2010, as well as the introduction of Competency-based Training (CBT) from the early 1990s, has led to an increased focus on outcomes in the Australian VET system. Industry- and company-specific training packages were designed to complement further training offers, thus creating opportunities to take greater account of the individual and demand-related needs of employees (Malloch and Helmy 2015).

The focus on the skills and abilities of employees has thus led to a differentiation of training programmes for all age groups, since these have now become more labour market-oriented and modularised. This development has also resulted in greater flexibility in the training programmes (Deißinger et al. 2017). In addition, the standardisation and modularisation of the initial and continuing vocational training system has made it possible to achieve a smooth transition between the various offerings of the Australian education system. The restructuring of the VET system is clearly reflected in an increase in the participation rates of the various VET programmes across all age groups (CEDEFOP 2009; Hoeckel et al. 2008; Struthers and Strachan 2019).

In Australian economic statistics, education exports rank third in terms of export earnings, making education as one of the top export of services in Australia. A distinction is made between *offshore* and *onshore* delivery, which differentiates between actual exports to various destination countries and the provision of educational services to learners from abroad in the home country (Fraunhofer MOEZ 2012). Australian regulatory frameworks state that no matter where an Australian VET course is undertaken, including offshore offers, the standards must be similar to those in Australian settings (Dempsey and Tao 2017). Australia's geographical proximity to countries such as China and India may benefit Australian education providers, as the demand for education in these countries has increased in recent years (Hall and Hooper 2008). In Tran and Dempsey’s (2017) study, there are several examples of countries (e.g. China, Kuwait, Vietnam) that raise demand for exports of Australian VET practices. Offshore offerings of TAFE (independent public VET providers) constitute more than 70% of Australia’s offshore vocational education (Dempsey and Tao 2017)—this highlights the relevance of the sales to foreign customers.

With all these requirements, the Australian TVET sector is geared to be flexible, with multiple pathways to and from the Australian Qualifications Framework (AQF; Unesco-Unevoc 2018). Qualifications can be undertaken at school, in the workplace and in registered training organisations (RTOs).

States/territories are responsible for the accreditation of courses, and they control and monitor public and private providers of TVET offers. Such providers need to fulfill national standards to be RTOs, and then they are allowed to offer nationally accepted courses and qualifications (Deißinger et al. 2017). Different kinds of certificates (certificates I–IV; diplomas; advanced diplomas; associate degrees) at different ISCED levels (2–5) lasting between 6 months and 4 years and can all be taught by private training providers (Unesco-Unevoc 2018).

The percentage of students undertaking VET with private training providers in Australia was 66.3%, thereby more than 3,000 private training organisations provided TVET (Unesco-Unevoc 2018).

There has been a significant increase in private contributions to the non-government school sector in recent years, where 20% of all expenditure on school education comes from private sources (Noonan et al. 2015). Of 4302 RTOs in 2019, 75% were private training providers. The remainder were publicly funded institutions, such as TAFE institutions, some dual-sector universities and schools or other providers, e.g. community education providers, enterprise providers and industry and professional associations (Joyce 2019).^4^

### The USA

The TVET system in North America is complex as it incorporates various grade levels, providers, and subject areas. This complexity comes from the fact that the decision-making regarding the framework of the TVET varies from state to state. In addition, it is supposed to be very dynamic because of the presence of many private training providers. Moreover, the market is described as being highly fragmented with the presence of many training providers (Technavio 2016a). End users have the option of choosing from a wide variety of TVET providers, including public-funded vocational schools and community colleges. Private training institutions and organisations are leveraging faculty with industry expertise from corporate training institutes and from massive open online courses (ibid.).

Within the US context, TVET is also called “Career and Technical Education (CTE), also known as career-tech. It describes a range of educational offers: TVET programmes are taught in non-parallel sub-systems, which take place in a public education system largely limited to high school, community or technical colleges, and they serve a wide range of public needs, government programmes, a miniscule apprenticeship system, *and a large business-based training system* disconnected from all of the others (Unesco-Unevoc 2014).

Apprenticeship programmes often target apprentices who have some years of experience. Community and technical colleges are the main providers of most apprenticeship programmes, while others are offered at employer-owned or employer-operated facilities and trade schools (Unesco-Unevoc 2014). The TVET market is internationalising its practices as a fundamental strategy for improving the sector: About 40% of the total international students enrolled are in community colleges; and VET is also being used as a pathway to enter higher education (Tran and Dempsey 2017). “In the USA, given their economic orientation, community colleges are highly adaptive and responsive to resource providers, whether states, private businesses and industry, or fee payers” (Levin et al. 2017, p. 20). Therefore, education policies and practices point to the ways in which neoliberal or liberal market ideology used the globalisation tendencies (ibid.).

In-company training represents a significant share of all TVET possibilities in the United States. It is provided by companies to their employees, and it is independent of governmental or educational pathways. For instance, the American Society for Training and Development declares that in-company training reveals a larger investment in TVET than that provided through public schools and colleges: It is within this business-based system that most occupational training and certification for workers takes place (Unesco-Unevoc 2014).

The National Skills Coalition of the US predicts that 52% of job openings from 2012 to 2020 will be in middle-skill areas, which require TVET instead of conventional education, such as high school diplomas or bachelor’s degrees (Technavio 2016a). Middle-skill jobs include technicians, police officers, electricians, firefighters, plumbers, welders, dental hygienists, respiratory therapists, and radiologic technicians. According to Technavio (2016a), job seekers should acquire the required specialised skills needed for such jobs. In order to obtain these skills, private training institutes are viable options. In 2015, the Science, Technology, Engineering and Math TVET segment was the highest contributor to the market in North America (ibid.).

### The UK

In 2006, the Leitch Report was published in Great Britain. This aimed to establish a training and further education system by 2020 to focus more on job-related skills and abilities in order to become more competitive worldwide. National prosperity is thus to be guaranteed by competence-based competitiveness, which, at the same time should enable individual adaptation to change processes (Leitch 2006). The government is fundamentally responsible for ensuring formal framework conditions and for intervening in market economy imbalances. The training system is to be made more demand-oriented by involving employers more closely. The aim is thus to increase qualifications and productivity, which is to be achieved fundamentally through a quantitative increase in skills. Some of the principles of Leitch were retained (CIPD 2017), but beyond that, in recent years there here has been a strong focus in England on improving the quality, widening the offer and increasing the uptake of apprenticeships (Department for Education 2021).

England, Wales and Northern Ireland differ only slightly in their education policies. Scotland, in contrast to the other three countries, focuses less on specialisation in its education policy and thus aims for broad content knowledge instead (Department for Employment and Learning 2011; Leitch 2006; Scottish Government 2010; Watson et al. 2020).

VET in the UK is available at secondary and higher education levels in the form of broad introductory courses and specialised advanced training (Cedefop 2017). The technical and vocational education system primarily focuses on learners who have left school and who have yet to enter the workforce or enroll in higher education, as well as employees who want to attain further sector-specific qualifications (Technavio 2016b).

For the UK, we state that although there has been a shift of financial responsibility towards the public, the setting of skill standards and assessments has been devolved to the sectoral level. Examples are the Sector Skill Councils with firms, the introduction of competency-based assessment with regard to National Vocational Qualifications in the 1990s and the retraining through a nationally standardised curriculum (Vossiek 2018). “The role of government in English skills formation is limited to create favorable market conditions for competing education providers to meet employer needs” (Esmond and Atkins 2020, p. 199). Therefore, educational standards vary strongly, which leads to huge variations in skill levels and standards between different sectors and to the volume of training being more important than the training quality (ibid.). This is consistent with the observation of Technavio (2016b): While students attained certifications and diplomas, many of those qualifications are not credible in the job market. The complex qualifications system in the UK makes it difficult for learners to assess the credibility and value of different qualifications.

There are numerous competing corporate training vendors in the UK market. The UK TVET system emphasises competition between providers (Baldauf et al. 2008); thus, the bargaining power of suppliers is high in the technical and vocational training market in the UK (Technavio 2016b). Training providers can be training or Human Relations (HR) departments in firms, further education colleges or private training organisations (Cedefop 2005). In 2018, there were more than 6500 private and employer training providers in the UK (Department for International Trade 2018). Baldauf et al. (2008) recognize that UK providers have a good reputation internationally regarding the quality of their education.

The British system appears to be very attractive due to the high participation of employers and the adaptation of the educational content. In addition, British education providers benefit from the high demand for English language courses. On the other hand, there is criticism of the low focus on the provision of general education and the lack of funding opportunities for public institutions, meaning that internationalisation efforts often do not take place because of too high risks. At the same time, this market-oriented approach means that the UK's education providers have a strong entrepreneurial approach to opening up new markets. Last but not least, this approach leads to strong competition among education providers (Baldauf et al. 2008).

In the UK, the export of vocational training can also be divided into *onshore* and *offshore* services. The offshore sector of VET exports in the UK is barely statistically accessible, which is why only data for the onshore sector are available. Thus, in the area of VET, GBP 138.6 million was generated by foreign learners in 2008/09, while British further education institutions were able to generate GBP 26.8 million from transnational education (Department for Business, Innovation and Skills 2011).

### Germany

Germany is considered to be one of the countries with the longest tradition of vocational education (Peters 2019). The Dual System of VET is the "the largest vocational Qualification Machine of the German Education System" (Greinert 2006, p. 499; translated by the authors). The system has, based on the resuscitation of a medieval-style system, developed over decades. The dual system is the result of a cooperation between different actors. It has been and is still based on a constant state of tension between the education system and the labour market (Michaelis and Busse 2021). Central to the development of the vocational training system is that no substantial connection to the academic institutions of higher education has emerged (Baethge et al. 2007).

Since the 1970s, the demand for training places has increased due to the baby-boomer generation. In connection with the expansion of education, an upgrading of the training took place along with the development of a high number of newly concluded training contracts. Up to 70% of the cohort took up vocational training (Baethge et al. 2007). Since about the beginning of the 1990s, the importance of education has decreased, and since then, there has been a clear trend towards studying and academisation (Alesi and Teichler 2013; Baethge et al. 2007).

*Continuing education* is offered by municipal institutions, especially by adult education centres (“Volkshochschulen”), and by *private institutions*, trade unions, various chambers of industry and commerce, political parties and associations, companies and public authorities, family education centres, academies, technical colleges (“Fachschulen”), Professional Academies, institutions of higher education and distance learning institutions (Unesco-Unevoc 2012).

The principles for the promotion and funding of continuing education are set out in the continuing education and employment legislation of the federal countries of Germany (“Länder”). The latter recognises the freedom in the preparation of curricula and independence in terms of staff selection of the continuing TVET providers (ibid.).

The landscape of providers in the field of continuing general and vocational continuing training in Germany comprises some 18,000 organizations, including all institutionalised or company-based providers that offer continuing training as a primary or secondary task on a regular or recurrent basis in an open and accessible manner, targeting trained or experienced adults (Christ et al. 2020). The business models of the continuing training service providers reflect the general heterogeneity of the sector. Overall, participants or self-payers (32%) and enterprises (24%) are the most important sources of funding, while 25% of the revenue from public funds comes from regional authorities (local, state, federal, the EU) and 14% from employment agencies and job centres. Private commercial providers generate 27% of their income from the contributions of participants or self-payers and 41% from corporate customers. Although education exports still account for a relatively low share of total economic exports, estimated at 0.1%, growth since 2010 has averaged 7.3% per year (WifOR 2018). According to a survey conducted by iMOVE of internationally oriented education providers, the share of education exporters in the German vocational training sector is estimated at 10% to 15%, which corresponds to an absolute number of exporting education service providers of around 2,500. An overwhelming majority of 70% of those surveyed assess the growth opportunities for export business as positive (iMOVE 2017).

Summing up, we see that there are very diverse market conditions in the four countries. The German system is characterised by a strong state-led education system and slender numbers of private education providers. However, there are exceptions (like Festo Didactics), and the market is expected to grow, and there are many instruments for the export promotion of VET services (Krichewsky-Wegener and Brings 2020). To collect views with a practical orientation in addition to perspectives from literature, we asked experts—recognised as such due to their professions as providers of VET services. Their responses to our guiding questions widened our view and sharpened our understanding of the market conditions.

## Results: Key feature services and strategies of TVET providers

Now that we have clarified the context of the research, we will deductively analyse of the conducted interviews. The research questions focused on the characteristics and conditions with regard to placing VET services and the success factors related to VET providers in international markets. We also consider relevant implications regarding German TVET providers in international markets.

Related to the analysis of the qualitative data, we firstly conducted a deductive round of coding, using Pilz’ (2016) criteria for analyses: *curricula*, *learning location*, *content*, and *learning process*. The results are presented below.

In terms of the *learning location*, we found that all different business models result in different locations. In our very diverse sample, all of the providers offer e-learning as a main product, and classroom learning is increasingly being substituted with e-learning and blended learning. This is not surprising, especially with regard to the current developments during the Covid-19 pandemic. More specifically, several VET providers cooperate with industrial partners, especially in relation to infrastructure-related vocations, e.g. chemical engineering, mechatronics, or robotics. Here, the curriculum is usually developed together with the industrial partner and the schools. These providers see their role as the didactic content partner because the learning location is either an educational institution (school, university) or online. Other providers use company placements or external training centres, which result in a kind of internship model where learners go directly to companies or training centres. At the same time, for employers, learning on the job is attractive, as they do not want “*to take employees off the assembly line for three days and lock them up in a workshop*” (I6) to do specific training. Furthermore, external private providers of blended and online study courses represent particularly interesting partners for VET providers looking to create e-learning.

With regard to the *learning process*, widely diversified business models can be seen, but the opinions about learning are quite consistent: “*Learning is actually independent of the educational system, which is going very much in the direction of personalised, adaptive learning”* (I2). This goes along with other statements that see asynchronous learning as being in demand: “*People simply want to learn on demand exactly what they need on the spot*” (I1). Generally, learning seems to be changing due to the facts of digitalization and free available learning contents (e.g. YouTube). Therefore, learning management systems are highly demanded. In terms of the learning process, in particular, we recognise that an adaption to the target market is important: For example, in China, the relationship between teachers and students is clearly a relationship of authority. Therefore, learning guidance is highly necessary in learning environments. The cultural habituation to technology in everyday life is also important when designing the learning process: Expectations regarding learning tools vary considerably depending on habits.

This leads us to the next criterion – *content*. The interviewees pointed out their type of educational service, and whether they structure content or develop content (e.g. audio, video or dynamic, interactive content). Mostly, a demand is recognised in terms of very specific professional training and further professional qualifications, and often for company-specific, rather than generic, vocational training, for example, preparatory content and courses for the entry examinations in professional fields like the police and fire brigade. Also, regarding such content, it is useful to look at the partnerships between VET providers and external partners: “*Of course we primarily offer the content, but we are more and more interested in finding partners in the field. This is a development that comes from the Anglo-Saxon world. […] So that's really a package of services that we're offering down the road*” (I5). For example, this provider offers partnerships for the design, implementation, operational management and marketing of online degree programmes. Furthermore, we observed how in countries that already have a high degree of social mobility, the kind of content that has a high degree of mobility works very well, such as that concerning nursing professions or IT. This is content that is demanded by many potential worldwide employers.

Last but not least, we found out that the *curriculum* is playing less and less a role from the perspective of VET providers. The reason is that the products “*depend very much on what the customers want. So, the market—and with the market we earn our money—does not ask for a curriculum at all”* (I1). Here, the interviewees observe a difference between Germany and the rest of the world: “*Learning goals in Germany are very much set in stone and are still very much based on a curriculum; elsewhere, this is not [the case]*” (I7). Still, although a curriculum within a product is not what the market wants, regulations and curricula are very important when designing products. The curriculum and the requirements are very specific, for example, in Germany. From the point of view of international VET providers, it is sometimes difficult to enter the German market with global solutions, especially if they have been developed in the Anglo-Saxon area. Therefore, for example, in the field of online and e-learning, the clothes of nursing staff must be white in Germany and not blue like in other countries. That does have an impact and means products cannot be translated or transferred directly. Here, the costs of transfer are obvious, and often it is not profitable to invest.

Given these criteria regarding VET-related products, the providers characterise their offers in the following ways. They either state that they only produce software (without content), or that they only produce content (without software). For both kinds of business, there is an expert partner (for the content) or a technical partner or implementation partner (for the software) who fills in the gaps.

In the inductive round of coding, we discovered that *markets* and *skills* were important topics. The issue of markets will be divided into sub-categories: *Anglo-Saxon* market characteristics, *access to markets* and *changing markets*.

Most of the interviewed providers operate worldwide (7 out of 9), and the others focus on local areas, like German-speaking countries. In general, the *Anglo-Saxon* market is characterised by a system of tuition fees; here, offers can be structured quite differently than in continental Europe where the university and college culture, in particular, is completely different. To meet this challenge, one market strategy of an interviewed provider is to use a very decentralised structure for international markets management and an entangled system of cooperation between employers and learning centres, along with clearly distinct market units (I9). This allows the market-specific adoption of products. Others are successful in recognising cultural differences: “*But, we believe there are often similarities in how training is designed*” (I3). For example, the U.S. market, with its large area and consistent language, provides a much bigger market for online study courses.

*Access to markets* is, in the cases of global corporations, a strategic decision. However, with small and medium-sized providers in the sample, it becomes clear that personal networks and contacts enable access to markets and, therefore, random encounters and opportunities. Additionally, the smaller companies interviewed see it as an advantage to be able to react quickly to current developments (e.g. the economic situation; I4); compared to large institutions, which, according to their reputations, tend to implement changes more slowly. At the same time, being as active as possible is often a challenge for the smaller ones. English being the native language of the USA, the UK and Australia is seen as a decisive competitive advantage by all the interview partners.

The topic of *changing markets* relates not only to the Covid-19 pandemic but also to changes in the world of learning. Digitalisation and distance learning entered the world of learning long before Covid-19. One illustrative development outlined by several interviewees is the market trend of gamification: Coming from the Asian region, the demand for gamification is increasing at the moment. Other general remarks are that individualisation, such as the motivation of learners, automated support, architecture, adaptive curricula or virtual reality (VR), are important developments in the training market. For one provider (characterised as a global market leader), we found that black markets are a current challenge of changing markets. Here, it is important to see the difference from our other interviewed providers: This global market leader uses a business-to-business (B2B) market strategy and, therefore, does not address private customers.

As well as market issues, *skills* were identified as being important by all the providers. An increased interest in the teaching of skills has been noted by most interviewees. Therefore, for example, modularised learning is in demand, and, “*the system—whether Anglo-Saxon or European or American – plays less and less of a role when we think in terms of skills. And the demand for this is increasing*” (I2). Despite all the cultural differences and their consequences, in terms of skills, VET-related services seem to be converging, regardless of the market.

Table 2 provides an overview of all the categories developed. It shows the incidence of the themes in the respective interviews.

**Table 2:** Categories and cases. Own compilation

Category	Sub-category	I1	I2	I3	I4	I5	I6	I7	I8	I9
*Learning location*	E-learning	x	x	x	x	x	x	x	x	x
	Cooperation (industrial partners)	x	x	x	x	x			x	x
*Learning process*	Personalised, adaptive learning	x	x	x	x	x	x	x	x	x
	Learning on demand	x	x	x	x	x	x		x	x
	Changing due to digitalisation	x	x	x	x	x	x	x	x	x
	Demand: learning management systems	x	x	x	x				x	x
	Adaption to target market	x	x	x	x	x	x		x	x
*Content*	Structuring content	x		x					x	x
	Developing content		x		x	x	x	x		
*Curriculum*	Adaption of products to regulations	x	x	x	x	x	x	x	(x)	x
	No (or rather few) curricular products	x		x	x		x	x		x
*Market*	Anglo-Saxon vs. others	x	x	x	x	x	x	x	x	x
	Access to markets (worldwide)	x	x	x		x	x	x		x
	Changing markets	x	x	x	x	x	x	x	x	x
*Skills*	Modularised learning	x	x	x	x	x		x	x	x
	Increasing interest in skills	x	x	x		x		x	x	x

**Fig. 2:**
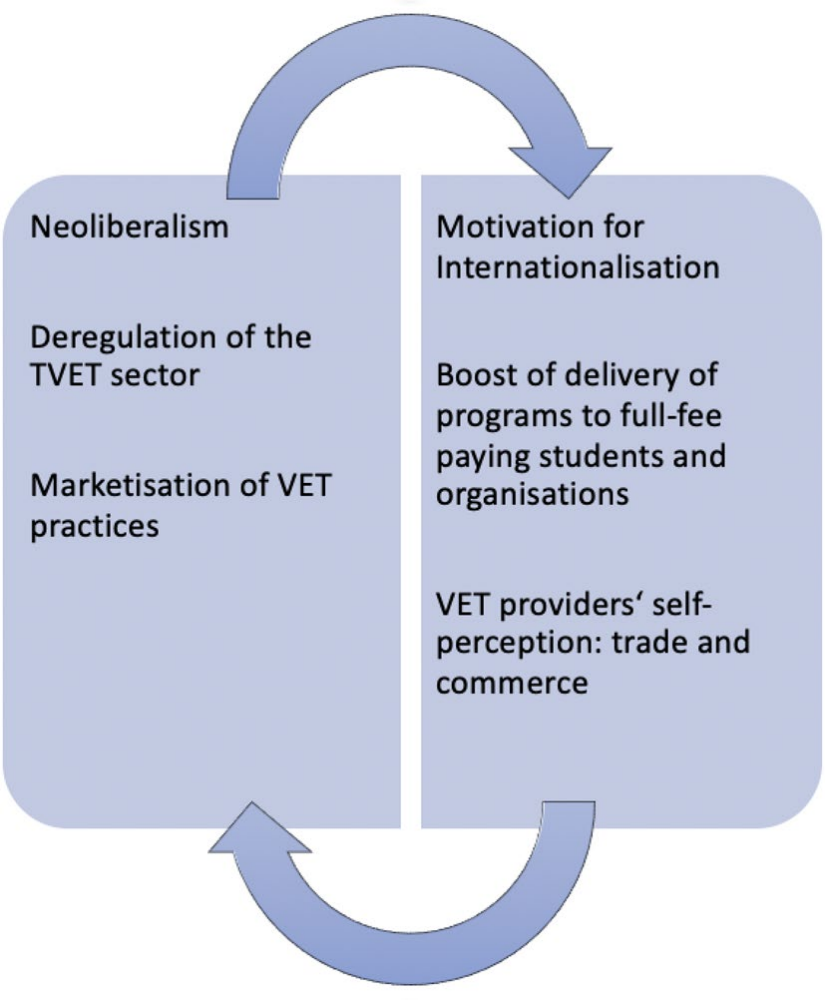
Symbiosis of findings. Own compilation

We conclude that our descriptions of the respective VET systems are in agreement with the findings of the qualitative content analysis. In the symbiosis (see Fig. 2), it becomes clear that neoliberalism and the deregulation of the TVET sectors (Pasura 2015) as well as the marketisation of VET practices (Tran and Dempsey 2017) lead to incentives to internationalise the VET offers. More and more full-fee-paying learners and organisations are available and the VET providers’ self-perception regarding their businesses can be described as trade and commerce (Pasura 2017). This goes along with the observation that the education system does not play a role—as long as you think in terms of skills. This is the case in liberal market-driven VET approaches and is mirrored in the micro-analysis categories (*curricula*, *learning location*, *content*, and *learning process*).

In Germany, this is not the case. Here, rather, a holistic and integrated development of knowledge and transferable skills for work and life make up vocational *education* (in contrast to vocational *training*; Pasura 2015).

## Discussion and conclusion

Returning to the research questions and which conditions and characteristics can be determined regarding the markets of VET-related services, as well as which success factors can be identified, we can say that Anglo-Saxon markets do have their own features that foster the provision of VET related-services. Also, access to markets and changing markets determine the provision of these services.

Above, we saw the different prerequisites of TVET systems in the respective countries. A focus on outcomes (Australia), a fragmented market with the presence of many training providers (the USA), a market-oriented approach to VET (the UK) and a generally open attitude towards commercial education (Australia, the USA, the UK) seem to be in contrast to the German cooperative approach in relation to the education system and the labor market and the clear distinction between initial VET and further education. In Germany, this leads to different providers’ self-perceptions and to internationalisation not being triggered.

This impression from the literature has been supported by the practical insights from our interviews: “*German providers come from the German perspective on education*”, meaning that in Germany, we see “*education as an integral good with Humboldt’s freedom of teaching*” (I2). Changes have also been seen in Germany regarding learning processes, and this may be described as a “technological pull effect” (I4; Dobricki et al. 2020). However, these developments are regarded as being slow, even cumbersome, in Germany: “*Particularly in the case of institutional education in Germany, decisions are often very lengthy processes, which is why we have more customers abroad than in Germany*” (I3). The key message of several interview partners is that this does not create an environment for private VET providers.

This leads us to a further question: What can be derived from this information regarding German TVET providers in international markets? Offering niche products, in the sense of the product focus rather than differentiation, seems to be a working strategy. Furthermore, we state that markets for VET-related services are successfully developing where state provision is rather weak, e.g. when the state provides a rather limited supply (the privatisation of the education system), or when the VET-related service is not covered by public educational offers (e.g. in highly specialised IT).

Language seems to be a disadvantage from the German perspective: English, as the native language of the USA, the UK and Australia, is a decisive competitive advantage. Also, regarding the products, modular learning opportunities, which are more common in the selected countries than in Germany, also represent a competitive advantage. Additionally, global market leaders operate in market units and, therefore, adapt their products to the target markets.

What does this mean for providers of TVET products? They need to keep in mind the changing form of enterprises. Previously, we have had service companies, on the one hand, and classical production companies, on the other hand.^5^ However, nowadays, production-*related* services (e.g. software production) are increasingly provided, and companies offer cross-sectoral (goods and related services) products at the same time. From our perspective, we also see this development in VET-related services. It is not clear whether providers offer initial VET or further VET—because in the market, it does not matter: “*If a customer chucks money at us, then I don’t ask whether I have to do VET or further education*” (I1). Although no final assessment can be made on the basis of the small number of cases, it can nevertheless be stated that the German form of VET therefore does not easily facilitate access to markets. In terms of education ideals, we do not bolster the market-driven approach, in our view, the state should ensure the provision of initial training as a meritorious, public good. In liberal VET markets, providers could adopt more dynamic, innovative approaches and integrate the expressed needs of individuals and organisations with the local socio-economic development agenda (Ramasamy and Pilz 2020).

Finally, we want to state clearly in contrast to our research desiderata, what our research was not about: Neither do we analyse a VET transfer (see e.g. Li and Pilz 2021) nor do we directly ask about the correlation between private training provision and the quality of training (Chankseliani and Anuar 2019; Toner 2018). It would be useful to study both approaches in future investigations, as well as the meaning of uniform certification models in internationalization of VET services. Regarding the limitations of the study, with such a small sample, we cannot generate generally valid results; also, the chosen method of qualitative content analysis can only limitedly analyse specific characteristics, such as development processes. Besides, researchers as well as interviewees always start out from their own context, which can skew the results. A large-scale quantitative survey would be advantageous in validating the results of our qualitative study.

## Data Availability

The qualitative data (audio files and transcripts) are available from the corresponding author on reasonable request.

## Footnotes

1 https://www.festo-didactic.com/int-en/.

2 https://www.udacity.com.

3 Australia has not been part of the sample due to field access; nevertheless, the large companies offer their training services in Australia as well and have been interviewed regarding that market.

4 https://www.ibisworld.com.au/industry-trends/market-research-reports/education-training/technical-vocational-education-training.html

5 In a complete classification, we would add “craft companies” as well, but for our purpose, this is not relevant.
